# EU regulation of gene-edited plants—A reform proposal

**DOI:** 10.3389/fgeed.2023.1119442

**Published:** 2023-02-14

**Authors:** Brigitte Voigt

**Affiliations:** Faculty of Law, Chair of Constitutional and Administrative Law, Public International Law, European and International Economic Law, University of Passau, Passau, Germany

**Keywords:** gene editing, reform, EU regulation, genetically modified plant, new genomic techniques

## Abstract

This article presents a proposal on how the European Union’s regulatory framework on genetically modified (GM) plants should be reformed in light of recent developments in genomic plant breeding techniques. The reform involves a three-tier system reflecting the genetic changes and resulting traits of GM plants. The article is intended to contribute to the ongoing debate over how best to regulate plant gene editing techniques in the EU.

## 1 Introduction: The need to update the EU’s GMO regime

**FIGURE 1:**
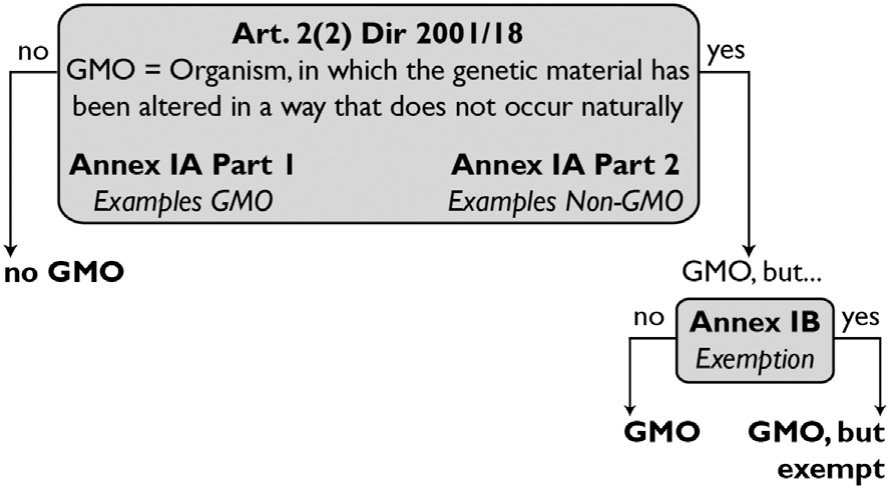
The EU’s definition of a GMO.

**FIGURE 2:**
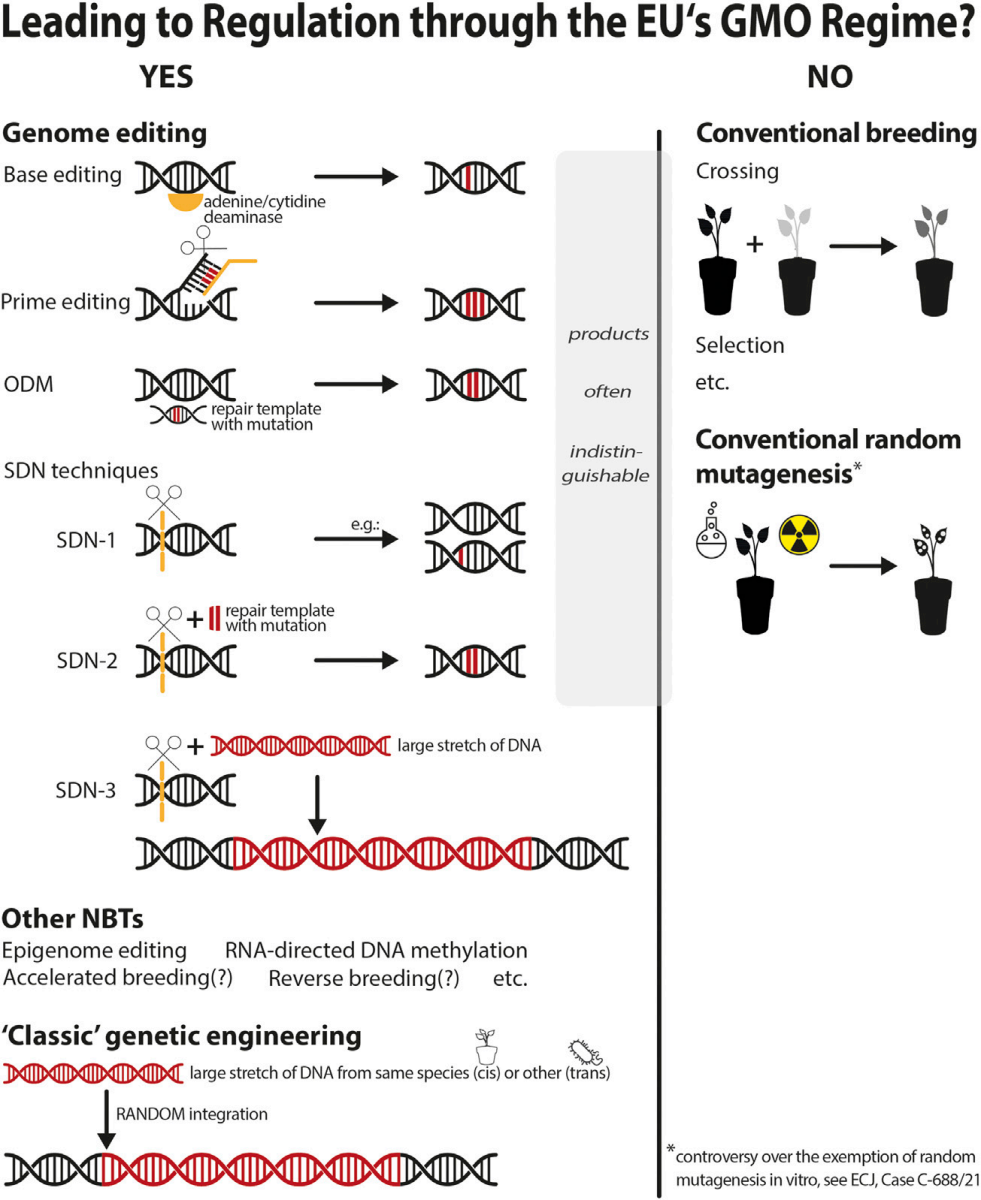
Status quo.

Within the last few decades several new genomic techniques (NGTs), also referred to as new breeding techniques (NBTs), have emerged, most prominently gene editing techniques that enable precise changes to be made to the genome. The latter include site-directed nuclease (SDN) techniques, which induce a double-strand break in the DNA and can be of type 1 (generating random mutations in precise locations), type 2 (generating a predicted modification in precise locations) and type 3 (inserting a large stretch of DNA in precise locations), oligonucleotide-directed mutagenesis techniques (ODMs), base editing techniques, prime editing techniques etc. (Broothaerts et al., 2021, 12–66; Molla et al., 2021; terminology differs between jurisdictions). The European Court of Justice ruled in 2018 that all gene-edited plants are regulated through the European Union’s GMO regime (ECJ, Case C-528/16 Confédération paysanne and Others [2018] ECLI:EU:C:2018:583, paras 47–48, 53; European Commission, 2021, 19–22; explanatory Figures 1, 2). This launched a debate over regulatory reform. Any reform must be effected at EU level, since the legislation on GMOs is, to a large extent, fully harmonised within the EU, meaning that no member state may apply more stringent or more lenient rules.

There is a need to amend the EU’s regulatory framework on GMOs where gene-edited plants are concerned, because some of these plants will only carry genetic changes that could also result from conventional breeding techniques. The current regulation of these plants under the strict GMO framework, without exceptions or simplifications, seems disproportionate, since it cannot be justified by reasons of precautionary health or environmental protection (see the findings in European Food Safety Authority, 2020, 2, 6; European Food Safety Authority, 2022, 19–20).

The current regulation is also impracticable. From the scientific point of view, there is currently no validated method of identifying gene-edited plants that carry only mutations that can occur naturally or can be obtained through conventional mutagenesis (European Network of GMO Laboratories, 2019, 7ff). Therefore, for these gene-edited plants and derived products the analytical control of the EU’s “zero tolerance” policy for unauthorised GMOs, as well as of the labelling of authorised GMOs, is difficult and in some cases simply not feasible (European Network of GMO Laboratories, 2019, 14ff, 17). From a normative point of view, requirements on the documentation of development, production and distribution could enable the required control, but this may introduce costs that are unreasonable and offend against the principle of proportionality.

In addition, the EU’s regulation of gene-edited plants is asymmetric vis-à-vis major trading partners of the EU, such as the UK (UK Parliament, 2022, at the time of writing not yet adopted), the United States, Argentina (and countries that employ the Argentine regulation of gene-edited plants as an example like Brazil, Chile, Colombia, Paraguay, Ecuador and Honduras), India (Government of India, 2022) and Japan (Dederer and Hamburger, 2019, as in 2018; Menz et al., 2020, 4ff). In these countries gene-edited plants, and derived food and feed products, that can also result from conventional breeding are largely unregulated through the relevant GMO regimes. This asymmetry entails costs, mainly for import-dependent industries like the feed industry, and might lead to trade disruption.

In April 2021, the European Commission published a study on new genomic techniques (European Commission, 2021). Drawing on the findings of this, it will propose a legal framework for gene-edited plants (more precisely: plants obtained by targeted mutagenesis and cisgenesis), and their food and feed products, in the **second quarter of 2023** (European Commission, 2022).

In what follows, a proposal for reform is presented to make an academic contribution to the ongoing debate on how to best regulate gene-edited plants in the EU.

## 2 Summary of the reform proposal

The EU’s definition of a GMO, and thus the scope of its GMO regulatory framework, will remain unchanged. However, within the GMO framework, a tiered regulatory approach will be introduced for **GM plants**, consisting of three levels of regulation:

**Tier 1:** For GM plants that could have been obtained by conventional breeding, including conventional random mutagenesis techniques, only a pre-market notification will be required, both for the release for experimental purposes and for introduction to the market as such or in products (including food and feed). The genetic modifications qualifying for Tier 1 will be exhaustively listed.
**Tier 2:** For GM plants and derived products that are not transgenic (and not covered by Tier 1), it will be decided on a case-by-case basis whether GMO authorisation is required. As part of the process of determining whether GMO authorisation is necessary (risk screening), the notifier will provide appropriate information allowing the competent authorities to evaluate whether there are indications that the GM plant, or its derived products, might pose a risk to human health or the environment. The evaluation will focus on the introduced/modified trait.
**Tier 3:** Transgenic GM plants will always need GMO authorisation.

In order to determine the organism’s status as a GMO, and the regulatory tier, a status procedure will be introduced.

The regulatory tiers are summarised in Table 1, and further explained in the following section.

**TABLE 1:** Overview of the reform proposal.

Tier	Applicability	Examples
**0:** Not regulated under the EU’s GMO framework	Plants not falling under the GMO definition (Dir 2001/18, art. 2(2)); GM plants exempted from the GMO framework (Dir 2001/18, Annex IB)	-Chemical and radiation mutagenesis
		-Null segregants
		-Epigenetic modifications
**1:** Notification	GM plants all of whose modifications are on a specified list – e.g. one constructed along the lines of US law: single modification that either (i) results from SDN-1 or (ii) is a single base-pair change or (iii) already occurs in the plant’s gene pool	-SDN-1 [single modification]
		-Base editing
		-SDN-2, ODM [introducing a naturally occurring genetic variation]
		-SDN-3 [allele replacement]
		*Tier 1 or 2, depending on the specification:* -SDN-1 [multiple modifications]
		-SDN-2, ODM [generating novel variation]
**2:** Case-by-case determination of whether authorisation is required (based on a risk screening)	Non-transgenic GM plants
		-SDN-2, ODM [multiple modifications]
		-SDN-3, ‘classic’ genetic engineering [cisgenesis; intragenesis]
**3:** Full GMO authorisation	Transgenic GM plants	-SDN-3, ‘classic’ genetic engineering [transgenesis]

## 3 Explanation of the levels of regulation

### 3.1 Tier 0: No regulation under the EU’s GMO framework

The EU’s GMO definition (arts. 2f., Annex IA, Annex IB Directive 2001/18, Figure 1) will remain unchanged. However, a guidance document containing legal interpretation will be developed interpreting the definition as follows: 1) an alteration of the genetic material in the sense of art. 2(2) Directive 2001/18 means only an alteration of the DNA sequence, i.e. epigenetic alterations are not alterations of the genetic material in the legal sense; 2) if the end product no longer contains the genetic alteration (the event), e.g. as is the case with null-segregants, it is not a GMO (its genetic material is not “altered” in the sense of art. 2(2) Directive 2001/18). Examples of null segregants include plants obtained by Accelerated breeding, and by Reverse breeding, since in these a foreign gene is integrated into the genome to induce early flowering and suppress recombination, respectively, but the gene is crossed out after the breeding process.

The general rules on the burden of proof require authorities prohibiting a non-authorised GMO/GM product to prove that the organism/product is indeed GM. However, a presumption rule will be introduced to facilitate market control: that is to say, it will be assumed that an organism is a GMO if techniques of genetic modification (i.e. those altering the genetic material in a way that does not occur naturally, see art. 2(2) Dir 2001/18, and that are not techniques in the scope of Annex IB Dir 2001/18) have been applied. Therefore, an organism will be treated as a GMO if it is not clear whether its genetic changes are a result of targeted mutagenesis or of conventional breeding (e.g. somaclonal variation). An example of an organism that would be presumed to be a GMO is the canola developed by Cibus, which has a mutation conferring herbicide tolerance (see below).

### 3.2 TIER 1: Notification

GM plants with genetic changes that could also occur naturally or be obtained through conventional breeding will require only a notification for field trials, for commercial cultivation of the plant, and for use in food and feed. Genetic changes of this kind will be **statutorily listed exhaustively**, making it straightforward to judge whether a GM plant belongs in Tier 1.

The genetic changes for which only a notification is required could be specified in one of two ways:

(i) along the lines of the US law [7 Code of Federal Regulations Part 340 § 340.1(b)]: “[...] plants that have been modified such that they contain […] a single modification of a type listed in paragraphs […] (1) through (3) of this section […]. (1) The genetic modification is a change resulting from cellular repair of a targeted DNA break in the absence of an externally provided repair template; or (2) The genetic modification is a targeted single base pair substitution; or (3) The genetic modification introduces a gene known to occur in the plant’s gene pool, or makes changes in a targeted sequence to correspond to a known allele of such a gene or to a known structural variation present in the gene pool.” (further US Department of Agriculture, 2022d; table of confirmation letters at US Department of Agriculture, 2022a)
(ii) more extensively: specification as (1) genetic modifications (of any number) resulting from cellular repair of a targeted DNA break in the absence of an externally provided repair template; or (2) modifications shorter than 20 base pairs; or (3) single modification known to occur in the plant’s gene pool, whereby (1)–(3) are exclusive alternatives, i.e. cannot be combined.

Depending on the specification, plants derived from such applications as SDN-2 and ODM would, or would not, fall into Tier 1.

Powers will be delegated to the European Commission to expand the list of genetic modifications in plants for which only a notification is required. The major prerequisite for adding a modification obtained by new genomic techniques to the list would be that sufficient experience with plants carrying the modification, including their deliberate release/introduction to the market, already exists. What would count as sufficient will need to be further specified by the EU legislator. The mechanism of gradually adding plants with additional genetic modifications will draw on US law [7 CFR § 340.1(b) (4); further US Department of Agriculture, 2020, 29794–29795]. Potential additions to the list of genetic modifications will include: 1) consecutive base pair changes up to a certain number; 2) identical modifications in more than two alleles of a gene in polyploid plants. Alternatively, a review clause could be introduced requiring the EU legislator to evaluate every 5 years whether the list of genetic changes for which only a notification is required should be updated.

**Information** that must be contained in the notification (specified in the implementing regulations) will include breeding and selection methods, the differences in genotype between the modified and the unmodified plant and a detailed description of the new trait(s) of the modified plant. It will also need to be demonstrated that the plant no longer contains foreign genetic material of the sort needed only during breeding, such as DNA coding for the CRISPR/Cas components. The required information would therefore be largely the same as that required for the status procedure (see below).

To meet the concern that the plant, or derived food and feed, might pose a risk to human or animal health, or to the environment, the authorities will use the legal instruments contained in the **general sectoral legislation** under the conditions laid down there (i.e. they will apply instruments that are used for conventionally bred plants and products as well). Examples here are the safeguard clauses under the seed legislation [e.g. Directive 2002/53, arts. 16(2), 18; Directive 2002/55, arts. 16(2), 18] and the emergency measures contained in the General Food Law Regulation (Reg 178/2002, arts. 53f.). Consequently, in practice, GM plants in Tier 1 would be **almost entirely deregulated**, and the primary purpose of the notification would be to inform the competent authorities about recent developments in plant breeding. Furthermore, the notification and subsequent publication of the notified plant in the GMO register will allow stakeholders rejecting gene editing—most obviously, the organic sector—to avoid them (freedom of choice).

### 3.3 TIER 2: Risk screening

For GM plants that are **not transgenic** (i.e. do not contain foreign genetic material derived from sexually non-compatible species) and not in Tier 1, it will be decided on a case-by-case basis whether GMO authorisations are required for field trials, for commercial cultivation of the plant, and for use in food and feed. Applicants wishing to avoid a GMO authorisation will need to demonstrate that there are no potential impacts on food/feed and environmental safety (see below). This risk screening is necessary because Tier 2-GM plants could not otherwise have been developed through conventional plant breeding techniques and might therefore have new, unfamiliar phenotypes. Applicants will also need to demonstrate that the plant no longer contains foreign genetic material of the sort needed only during breeding.

Decisions on whether GMO authorisation is required for field trials will be taken at the national level. By contrast, the decision on whether GMO authorisation is required for market approval will be taken at EU level (comitology procedure) after risk screening carried out by EFSA.

The **risk screening** could be designed as follows:

In order to demonstrate that an authorisation for **food and feed** is not necessary, the applicant will need to demonstrate that the composition, or structure, of the GM food, or feed, is not significantly changed in a way that affects its nutritional value, metabolism or level of undesirable substances. This requirement is identical, in its wording, with the definition of novel foods from plants in Regulation 2015/2283, art. 3(2)(a)(iv). It would need to be interpreted consistently. Applicants will need to assess the consequences of any intended compositional change. They will also need to analyse key nutrients, anti-nutrients, toxicants and allergens (see recommendations in OECD consensus documents, OECD, 2023), and compare them with ranges published for conventional varieties, e.g. in the ILSI Crop Composition Database and the scientific literature.

In order to demonstrate that authorisation for **field trials**, or **commercial cultivation** is not necessary, the applicant will have to demonstrate the familiarity of the genetic modification (i.e. the event) and/or the trait and underlying molecular mechanism. Information on the occurrence of the same/a similar genetic modification, as well as on the trait, and potentially the underlying molecular mechanism in plants of the same/a related species (including GM plants), would have to be provided. Differences (e.g. genetic modification exists in another plant species) will have to be assessed for their potential impacts on food and environmental safety (e.g. with a view to different wild relatives). In cases where the genetic modification is unfamiliar, the applicant will be required to show, with reference to the plant species, the trait and underlying molecular mechanism, that there are no reasons to believe that the plant would have adverse effects on human/animal health or the environment. In practice, the latter would be a simplified risk assessment, modelled on the *regulatory status review* in US law (7 CFR § 340.4; US Department of Agriculture, 2022e; see evaluations at US Department of Agriculture, 2022f). The required information could also draw on US law [7 CFR § 340.4(a)(4)].

The results of the case-by-case determination will be published to inform and facilitate future applications. Implementing acts will lay down details of, for example, the information required for the case-by-case determination and the conditions for asking for additional data.

A non-binding **preliminary determination** of the requirement for a GMO authorisation could already be obtained during the research and development stage of the GM plant.

In sum, the case-by-case determination is based mainly on the altered trait, and it is therefore a step towards a **product-based approach**. At the same time, it is a test of whether such an approach is viable in the EU. Once the EU has gained sufficient experience with Tier 2, an automatic exception mechanism could be introduced for combinations of plant, trait and underlying molecular mechanism that have already been evaluated, drawing on the US regulation of GMOs [7 CFR § 340.1(c)].

### 3.4 TIER 3: GMO authorisation

For the remaining — i.e. transgenic — plants, GMO authorisation will always be required. In cases of doubt whether gene transfer is possible or not, the procedure for determination of regulatory status would have to be followed.

## 4 Procedure for determining regulatory status

A procedure for determining GMO status and the applicable tier will be set out. This could draw, for example, on the procedure for determining novel food status under Regulation 2015/2283, arts. 4–5. The information required could mirror that required in existing GMO status procedures in third countries, e.g. Argentina, Resolution No. 21/2021, Annex III, or the US (US Department of Agriculture, 2022d, 6–7). No margin of discretion would be left to the competent authorities in determining the regulatory status.

Only the **intended genetic modifications**, i.e. those resulting in alteration of the trait, would be decisive for GMO status. No account would be taken of potential **unintended modifications**, e.g. off-target mutations, because gene-edited off-target mutations are fewer than the mutations occurring in random mutation breeding in plants, and detrimental mutations are reduced during the breeding process (European Food Safety Authority, 2020, 9–10). On the other hand, applicants will need to demonstrate that no foreign DNA (e.g. DNA coding for the CRISPR/Cas components) remains, however unintentionally, in the final plant, since the presence of such residual foreign DNA would be an unintended effect that cannot occur in conventional plant breeding.

## 5 Cross-cutting aspects and complementary reforms

**Other aspects of the GMO legislation** addressing risks of GMOs to human/animal health and the environment, in addition to authorisation requirements (e.g. monitoring under Directive 2001/18, Annex VII), apply to neither Tier 1-GM plants nor Tier 2-GM plants not requiring GMO authorisation.

By contrast, the opportunity for member states to **opt out** of GMO cultivation, under Directive 2001/18, art. 26b, would be retained for all GM plants. This means that member states can ban commercial cultivation of GM plants of all tiers on their territory under the opt-out process. The reason for this is that the opt-out clause concerns, not GMO risk regulation, but socioeconomic impacts, agricultural policy objectives, etc.

Traces of unauthorised non-transgenic GM plants, and derived food and feed, will be acceptable below a certain threshold (***low level presence***). This is designed to avoid trade disruptions, since in practice these traces will be almost unavoidable in export goods from third countries not regulating all gene-edited plants under their GMO framework, and as mentioned, analytical controls are not always feasible.

The tiered approach allows the regulation of plants obtained by new genomic techniques to be relaxed while the single GMO definition for all types of organism (plants, animals, microorganisms) is upheld. It does not mean that the regulatory framework for **animals and microorganisms obtained by new genomic techniques** is also relaxed. That may or may not happen at a later stage.

This proposal **does not address** the question whether all gene-edited plants, and derived food and feed, should be subject to **labelling, traceability and coexistence measures**.

## 6 Case studies

### 6.1 Soybean with altered oleic acid content obtained by SDN-1

A soybean line with increased levels of oleic acid and decreased levels of linoleic acid as a result of deletions in two FAD2 genes introduced *via* SDN-1 (TALEN) has been developed by the biotech company Calyxt (US Food and Drug Administration, 2019a; US Food and Drug Administration, 2019b; Health Canada, 2022). It is already marketed in the US.

Depending on how, exactly, the genetic modifications qualifying for Tier 1 (i.e. pre-market notification) are defined, the gene-edited soybean would fall into Tier 1 or Tier 2. If it falls into Tier 1, the applicant will be required merely to deliver a narrow information set, mainly comprising the information that is necessary for the determination of the soybean’s regulatory status. After that, and the expiration of a short waiting period, the soybean could be marketed throughout the EU: it could be cultivated commercially, soybean oil produced from it could be sold, etc. If the soybean falls into Tier 2, the competent authority (member states where field trials are concerned, the European Commission where introduction to the market is concerned) would need to determine whether full GMO authorisation is required. In this procedure, where import of food and feed into the EU is concerned, the applicant would have to demonstrate that the changes in the composition of the food (altered fatty acid profile of the oil) does not negatively affect its nutritional value, metabolism or level of undesirable substances. Here, the applicant could draw on the safety and nutritional assessment already presented to the authorities in the US and Canada.

### 6.2 Herbicide tolerant canola obtained by ODM

Cibus has developed a canola with increased tolerance to certain herbicides using an ODM method (on this and the following, see Health Canada, 2016). The canola possesses a specific single nucleotide mutation in the BnAHAS1C gene. It is unclear whether this resulted from a spontaneous somaclonal variation that occurred during the tissue culture process or as a result of the oligonucleotide used in the RTDS protocol. The canola is marketed in the US and Canada.

Under the legal presumption explained above, it would be assumed that the canola is a GM plant, since GM techniques have been applied. However, the alteration of a single nucleotide would only need to be notified (Tier 1). After the pre-market notification, the canola plants could be grown in the EU, and used for food and feed, without having to comply with GMO law.

### 6.3 Other examples

Other examples illustrating the applicable tier can be found in Table 2.

**TABLE 2:** Examples illustrating the applicable tier.

Plant	Trait	Technique	DNA change	References	Tier	Explanation	Remarks
Oilseed rape	Tolerance to ALS-inhibiting herbicides (Imidazolinones)	*In vitro* random mutagenesis (ENU)	1 base pair substitution in ALS1 and ALS3 genes	Swanson et al. (1989); Haut Conseil des Biotechnologies (2020), 26–27	Tier 0	Exempted from the GMO framework (Annex IB(1) Dir 2001/18)	Marketed as Clearfield canola
Rice	Tolerance to ALS- inhibiting herbicides	Base editing (CBE)	1 base pair substitution in ALS1 gene	Bioheuris Inc (2022)	Tier 1	Single base-pair change, see Section 3.2 of this article, (i)(2) or (ii)(2)	United States: exempt from regulation under 7 CFR part 340 US Department of Agriculture (2022c)
Soybean	Altered seed morphology and composition	SDN-1	Deletion in one gene (Result: gene knock-out)	Benson Hill Inc (2022)	Tier 1	See Section 3.2, (i)(1) or (ii)(1)	United States: exempt from regulation under 7 CFR part 340 US Department of Agriculture (2022b)
Tomato	Increase of γ-Aminobutyric acid (GABA)	SDN-1	1 base pair insertion in genes SIGAD2 and SIGAD3 (Result: gene knock-out)	Lee et al. (2018) and Nonaka et al. (2017)	Tier 2 or Tier 1	Tier 2, if genetic modifications for which only a notification is required are specified as in Section 3.2 (i) of this article (two genes are modified → not a *single* modification, i.e. alterations on one pair of homologous chromosomes); Tier 1, if specified as in Section 3.2 (ii), see (ii)(1)	Japan: marketed; United States: not regulated under 7 CFR part 340 US Department of Agriculture (2020)
Tomato	RNA interference mediated virus resistance	Intragenesis (particle bombardment)	Insertion of tomato derived DNA fragments	Maagd et al. (2020), 15	Tier 2	Not in Tier 1 (see Section 3.2 of this article); not in Tier 3 (entirety of the introduced DNA originated from the tomato genome)	United States: not regulated under 7 CFR part 340 US Department of Agriculture (2019)
